# Gastric Outlet Obstruction Due to Neurofibromatosis: An Unusual Case

**DOI:** 10.4103/1319-3767.45063

**Published:** 2009-01

**Authors:** Rajul Rastogi

**Affiliations:** Yash Diagnostic Center, Yash Hospital and Research Center, Civil Lines, Kanth Road, Moradabad, Uttar Pradesh – 244 001, India

**Keywords:** Gastric outlet obstruction, neurofibroma, von Recklinghausen

## Abstract

Neurofibromatosis type-1 (NF-1), also known as von Recklinghausen disease, is an autosomal dominant condition with an approximate incidence of one in 3000 births. NF-1 is known to involve multiple systems in the body. Abdominal involvement include neurofibroma and tumor growth in the liver, mesentery, and retroperitoneum in addition to gastric and bowel tumors. Gastrointestinal neoplasms occur in up to one quarter of patients. The author reports a rare case of diffuse submucosal neurofibromatosis resulting in gastric outlet obstruction.

Neurofibromatosis (NF) is a genetic, neurocutaneous syndrome categorized into clinically and genetically distinct two types: NF-1 and NF-2. NF-1, the peripheral type, is the most common type and is associated with abnormal skin pigmentation along with multiple nerve tumors, hamartomas, and other disseminated tumors. NF-2, the central type, is characterized by central nervous system tumors, especially the bilateral neurofibroma of the 8th cranial nerve. In the gastrointestinal (GI) tract, NF-1 is associated with neurofibroma, leiomyoma, and adenocarcinoma in the stomach, small and large bowel, vasculopathy, bleeding, pseudoobstruction, and protein-losing enteropathy.[1,2]

## CASE REPORT

**Figure 1:**
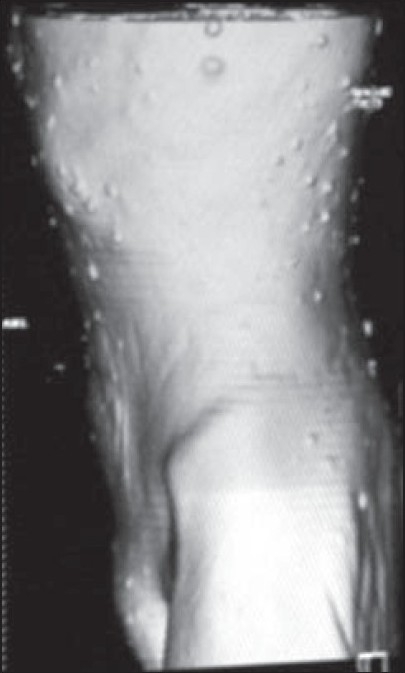
Surface-rendered CT image showing multiple cutaneous neurofibromas

A middle-aged female patient was referred to us for contrast-enhanced computed tomography (CT) of the abdomen. The patient had a history of recurrent vomiting for 3–4 months that was increasing in frequency. In addition, there was distension of the abdomen, especially after meals. The patient was known to have NF-1 and a clinical examination was remarkable only for the presence of multiple, cutaneous neurofibromas [Figure 1]. A recent ultrasound examination of the abdomen revealed a dilated stomach with no evidence of lymphadenopathy; upper gastrointestinal (GI) tract studies were not available.

**Figure 2:**
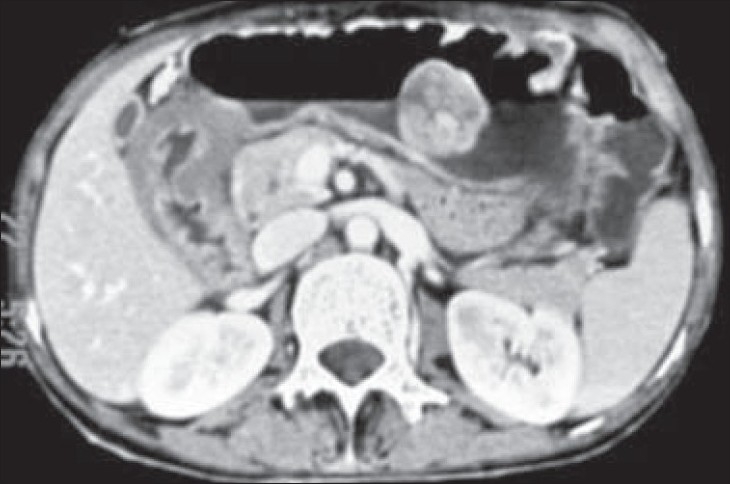
Axial contrast-enhanced CT image showing polypoidal neurofibroma along the greater curvature of the stomach and diffuse mural thickening in the region of pyloric antrum

**Figure 3:**
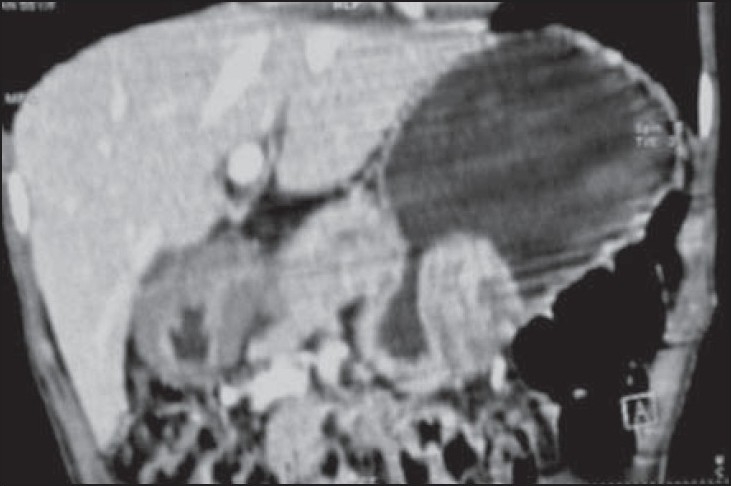
Oblique coronal CT MPR image showing polypoidal neurofibroma along the greater curvature of the stomach along with diffuse thickening of the wall of the pyloric antrum

Contrast-enhanced CT revealed a large, sessile, polypoidal, soft tissue mass with mild to moderate postcontrast enhancement along the greater curvature of the stomach. In addition, there was diffuse mural thickening of the pyloric antrum with overdistension of the stomach and delayed gastric emptying [Figures 2 and 3]. The thickened wall did not reveal any postcontrast enhancement and there was no associated lymphadenopathy. Based on the clinicoradiological findings, the provisional differential diagnosis of gastric adenocarcinoma and submucosal neurofibromatosis in the gastric antrum causing gastric outlet obstruction and possible neurofibroma along greater curvature was suggested.

The patient underwent surgery for the relief of the gastric outlet obstruction. Histological results of the polypoidal mass were consistent with those for neurofibroma, and the mural thickening in the pyloric antrum was found to be due to submucosal neurofibromatosis.

## DISCUSSION

NF-1 is a multisystemic disorder that may affect any organ in the body; the clinical presentation depends on the body system involved. GI tract tumors in NF-1 occur most frequently followed by thoracic tumors, arterial involvement, and endocrine tumors.[3,4] Abdominopelvic involvement is primarily extraperitoneal in NF-1.[5] GI tract involvement includes neurofibromas within the liver, mesentery, retroperitoneum, stomach, the small and large bowel, and the rectum. Small bowel leiomyomas, adenocarcinoma with neuroendocrine function, intestinal vasculopathy, bleeding, pseudo-obstruction, and protein-losing enteropathy can also occur. Patients may present with abdominal pain, nausea, abdominal distension, diarrhea, constipation, bowel perforation, or GI tract bleeding.

In one study[6], the majority of the neoplasms were located in the small intestine (72%). Neurofibromas found in 52% of the patients, were the most frequently diagnosed benign neoplasms, followed by leiomyomas (13%), ganglioneurofibromas (9.8%), and gastrointestinal stromal tumors (GISTs) (6.5%). Adenocarcinoma was present in 23% of the patients. Neurofibromas may be diffuse and submucosal with Auerbach plexus being the site of origin. Benign neurofibromas undergo malignant transformation in 5–15% of the patients, especially in patients older than 40 years.

Pathologically, neurofibroma in NF-1 patients may be of the localized, plexiform, or diffuse type. Localized neurofibroma appears as a nonencapsulated, well-defined tumor involving a single nerve and is composed microscopically of Schwann cells and fibroblasts with a myxoid or mucinous matrix surrounded by collagenous tissue with mast cell infiltration. Plexiform neurofibroma involves multiple nerve bundles or a plexus of nerve and shares the same histological features as localized neurofibroma except for higher cellularity and collagen. Diffuse neurofibroma is commonly seen in the head and neck region and appears as plaque-like lesions on the skin with the characteristic presence of fat seen by microscopy. The majority of patients with gastrointestinal neurofibromatosis have localized or plexiform neurofibroma along with diffuse thickening of the myenteric plexus. The presence of mitotic activity may be indicative of malignant degeneration.[7,8]

Immunostaining for S100 protein and neurofilaments is usually positive in neurofibroma due to the presence of residual myelinated nerve fibers. Sometimes, factor XIIa and cluster of differentiation 34 (CD34) positive cells may also be seen. Plexiform neurofibroma may be positive for epithelial membrane antigen (EMA) due to the presence of perineural cells. human melanoma black -45 (HMB45)- and melan A-positive cells may be seen in pigmented neurofibroma. The presence of S100 protein distinguishes neurofibromas from intramuscular myxomas. Immunostaining that is positive for CD34 and negative for S100 distinguishes a dermatofibrosarcoma protuberance from diffuse neurofibroma. Plexiform schwannoma is distinguished from plexiform neurofibroma by the presence of vimentin and myelin basic protein in addition to being strongly positive for the S100 protein.[8]

Imaging plays an important role in the diagnosis, evaluation, and follow-up of patients with abdominal manifestations of NF-1. Barium studies may show intraluminal mass lesions, associated intussusception of the bowel, or features consistent with malabsorption. A solitary neurofibroma is mostly hyperechoic on ultrasonography with coarse internal echoes, and although lobulated, it has smooth and well-defined margins. Plexiform neurofibromas appear homogeneously or heterogeneously hypoechoic.

CT scans demonstrate solid, intraluminal or intramural, unifocal or multifocal, spherical masses with central areas of low attenuation and occasional calcification. The masses usually are well-defined and have homogeneous low attenuation, equal to or slightly more than water but lower than muscles or adjacent soft tissues.[9] They may show mild to moderate, heterogeneous postcontrast enhancement. Sometimes, CT may reveal long segments of nodular mural thickening of the GI tract with with a variable degree of luminal compromise.[8] MRI is considered to be the modality of choice and may reveal several rings in T2W images and postGadolinium T1W images corresponding to a rope-like gross appearance in plexiform neurofibromas.[10]

Yoshinori *et al*. reported a case with a large gastric neurofibroma growing extramurally from the greater curvature of the stomach.[11] Halkic *et al*. reported a case of gastric neurofibroma in an NF-1 patient, causing gastrointestinal hemorrhage and severe anemia.[12] A rare case of mural gastric neurofibromatosis resulting in gastric outlet obstruction and subsequent perforation in an NF-1 patient has been reported previously only by Bakker *et al*.[6] To our knowledge, the present case represents the second instance where neurofibromatosis has involved the gastric wall with subsequent gastric outlet obstruction. However, in our case, early suspicion raised by preoperative CT prevented any subsequent complication of gastric perforation.

Because of the high likelihood of malignant lesions and high incidence of malignant degeneration in benign tumors, surgery is the preferred treatment of symptomatic intestinal tumors in NF-1 although patients without symptoms can be followed up nonsurgically.[13]
